# Population characteristics of children with short stature and construction of a predictive model for growth hormone treatment efficacy

**DOI:** 10.3389/fmed.2026.1818279

**Published:** 2026-07-07

**Authors:** Chuanwei Ban, Juan Wang, Qian Zeng, Ping Liu, Xin Lv

**Affiliations:** 1 Clinical Laboratory, Children's Hospital Affiliated to Shandong University, Jinan, China; 2 Clinical Laboratory, Jinan Children's Hospital, Jinan, China

**Keywords:** efficacy, growth hormone, predictive model, short stature, treatment

## Abstract

**Background:**

This study aims to analyze clinical characteristics and treatment patterns among children with short stature, and to develop a predictive model for growth hormone treatment efficacy for clinical reference.

**Methods:**

This was a retrospective cohort study including 400 children diagnosed with short stature. Patients were divided into three groups: growth hormone intervention group, nutritional support group, and untreated (watchful waiting) group, based on parental choice after clinical evaluation. Annualized growth velocity was compared between the growth hormone intervention and nutritional support groups. Within the growth hormone intervention group, 70% of subjects were randomly assigned to a training set and 30% to an internal validation set. Multivariate logistic regression was used to identify factors associated with growth hormone treatment efficacy. A nomogram prediction model was constructed and evaluated using receiver operating characteristic (ROC) curves, calibration curves, Hosmer-Lemeshow test, and decision curve analysis.

**Results:**

A total of 293 children were included after excluding 107 cases (76 with missing data, 11 with skeletal dysplasia, 16 with hypothyroidism, 4 with chromosomal abnormalities). The growth hormone intervention group (*n* = 124) showed significantly higher annualized growth velocity than the nutritional support group (*n* = 124) across all age groups (*p* < 0.05). Multivariate logistic regression revealed that duration of growth hormone therapy was the independent predictor of treatment efficacy (OR = 4.45, 95% CI: 1.53–12.93, *p* = 0.006). Six variables (treatment duration, age at initiation, insulin-like growth factor-1 (IGF-1), target height, baseline height, gender) were included in the nomogram model. The model showed good discrimination [area under the curve (AUC) = 0.88 in training set, 95% confidence interval (CI): 0.80–0.96; AUC = 0.85 in validation set, 95% CI: 0.69–1.00], good calibration, and favorable clinical net benefit.

**Conclusion:**

This study confirms that growth hormone treatment improves growth velocity in children with short stature. A predictive model was established and internally validated. Since treatment duration was the significant predictor and is observed during treatment, the model is more suitable for intra-treatment efficacy evaluation rather than pure pre-treatment decision-making. This model has the potential to support clinicians with individualized dynamic efficacy assessment and subsequent patient management, facilitating the optimization of follow-up treatment regimens.

## 1 Introduction

During a child's growth, parents frequently express concern regarding their children's height, given its implications for their nutrition and overall progress. Short stature can be caused by a variety of factors, including growth hormone deficiency (GHD), idiopathic short stature (ISS), familial or constitutional delay of growth and puberty, small for gestational age (SGA), thyroid disorders, chromosomal abnormalities, and skeletal dysplasia (1). Generally, short stature is defined as a height below 2 standard deviations (−2SD) or beneath the 3rd percentile compared to the average height of healthy children of similar age, gender, and ethnicity in a comparable setting (2, 3). Research in China indicates that approximately 3.2% of children suffer from short stature, highlighting the prevalence of this condition among children (4). The impact of short stature extends beyond physical health [such as respiratory function (5), blood lipids (6), and cardiovascular health (7)] to also affect mental wellbeing (3, 8). Although short stature has diverse etiologies, evidence demonstrates that growth hormone treatment achieves consistent and significant therapeutic benefits across different causes of short stature (9, 10), such as GHD and ISS. However, long-term growth hormone treatment imposes substantial economic burden, and reliable prediction of growth hormone efficacy remains challenging in clinical practice. This study retrospectively analyzed clinical data and treatment patterns of children with short stature. We further explored factors related to growth hormone response and developed a predictive model to support individualized dynamic efficacy assessment and subsequent management, balancing height benefits and economic costs.

## 2 Materials and methods

### 2.1 Study population

A cohort of 400 pediatric patients with short stature who visited Children's Hospital affiliated to Shandong University in 2025 was enrolled. The ‘Height and weight standardized growth charts for Chinese children and adolescents aged 0 to 18 years' serve as a reference for diagnosing short stature (11). Inclusion Criteria: Height below −2 standard deviations or below the 3rd percentile compared to the average height of healthy children of the same age, gender, ethnicity, and similar environmental conditions. Children with skeletal dysplasia (osteochondrosis), hypothyroidism, chromosomal abnormalities (e.g., Turner syndrome), and those with missing baseline information (such as gender and age) were excluded. This study was conducted in accordance with the Declaration of Helsinki and received approval from the Research Ethics Committee of Children's Hospital Affiliated to Shandong University (Approval No.: SDFE-IRB/P-2023061). This was a retrospective study. All analyses were performed using anonymized clinical data in compliance with relevant regulations and ethical standards. The requirement for written informed consent was waived by the Ethics Committee of Children's Hospital Affiliated to Shandong University.

### 2.2 Data collection and group assignment

From the hospital information system, detailed information was collected from the children's historical electronic medical records, including gender, age, height, and laboratory test results [such as hemoglobin (Hb), total cholesterol (TC), Calcium (Ca), zinc (Zn), vitamin D, insulin-like growth factor-1 (IGF-1), insulin-like growth factor binding protein 3 (IGFBP3)]. Children who met the exclusion criteria or had missing key information were excluded from the cohort. After patients were diagnosed with short stature, clinicians formulated individualized treatment plans in accordance with clinical guidelines. Since pediatric patients were too young to make medical decisions on their own, their parents made treatment choices after receiving detailed professional counseling. The treatment plan may include watchful waiting, nutritional support therapy, and growth hormone intervention. The untreated group received conservative observation, and the nutritional support group was supplemented with milk, eggs, and vitamin D daily as prescribed. The growth hormone intervention group received growth hormone therapy in accordance with the Guidelines for the Diagnosis and Treatment of Children with Short Stature in China, with an initial dose of 0.1 U/(kg·d), combined with routine nutritional support. All patients strictly followed clinical treatment instructions and attended scheduled hospital follow-up visits. Treatment regimen adjustments were made only after assessment of height growth status at each follow-up. We first compared the differences in annualized growth velocity between the nutritional support group and the growth hormone intervention group. Subsequently, we compared the impact of growth hormone intervention on growth velocity. 70% of participants in the growth hormone intervention group were randomly assigned to the model training set. Height standard deviation score (HtSDS) is an indicator that evaluates the degree of difference between an individual's height and the height of peers of the same age and gender. HtSDS equals measured height minus median height, divided by the standard deviation of height in the reference population. According to the “Height and weight standardized growth charts for Chinese children and adolescents aged 0 to 18 years” and the standards from referenced literature, we adopted change in height standard deviation score (ΔHtSDS) as the reference indicator for therapeutic efficacy. ΔHtSDS refers to post-treatment HtSDS minus pre-treatment HtSDS. Participants with 0.3 < ΔHtSDS < 1.0 were classified as the treatment-effective group, while those with ΔHtSDS ≥ 1.0 were defined as the treatment-highly-effective group (12, 13). We performed logistic regression to identify factors affecting treatment efficacy and constructed a prediction model for growth hormone therapy outcomes. The model was assessed for discriminative ability using receiver operating characteristic (ROC) curves, for calibration using the Hosmer-Lemeshow goodness-of-fit test, and for clinical value using decision curve analysis. Internal validation was conducted using the remaining 30% of participants.

### 2.3 Statistical analysis

All statistical analyses were conducted with SPSS and R software. Quantitative data were analyzed using *t*-tests, while categorical data were assessed with chi-square tests. Multivariable linear regression analysis was performed to address selection bias. The variance inflation factor (VIF) was used to assess multicollinearity among independent variables. Variables demonstrating statistical significance (*p* < 0.05) in univariate analysis were identified as independent variables and incorporated into multivariate logistic regression analysis to investigate factors influencing growth velocity. A predictive model was established using logistic regression through forward stepwise selection.

## 3 Results

### 3.1 Characteristics of study participants

**Figure 1:**
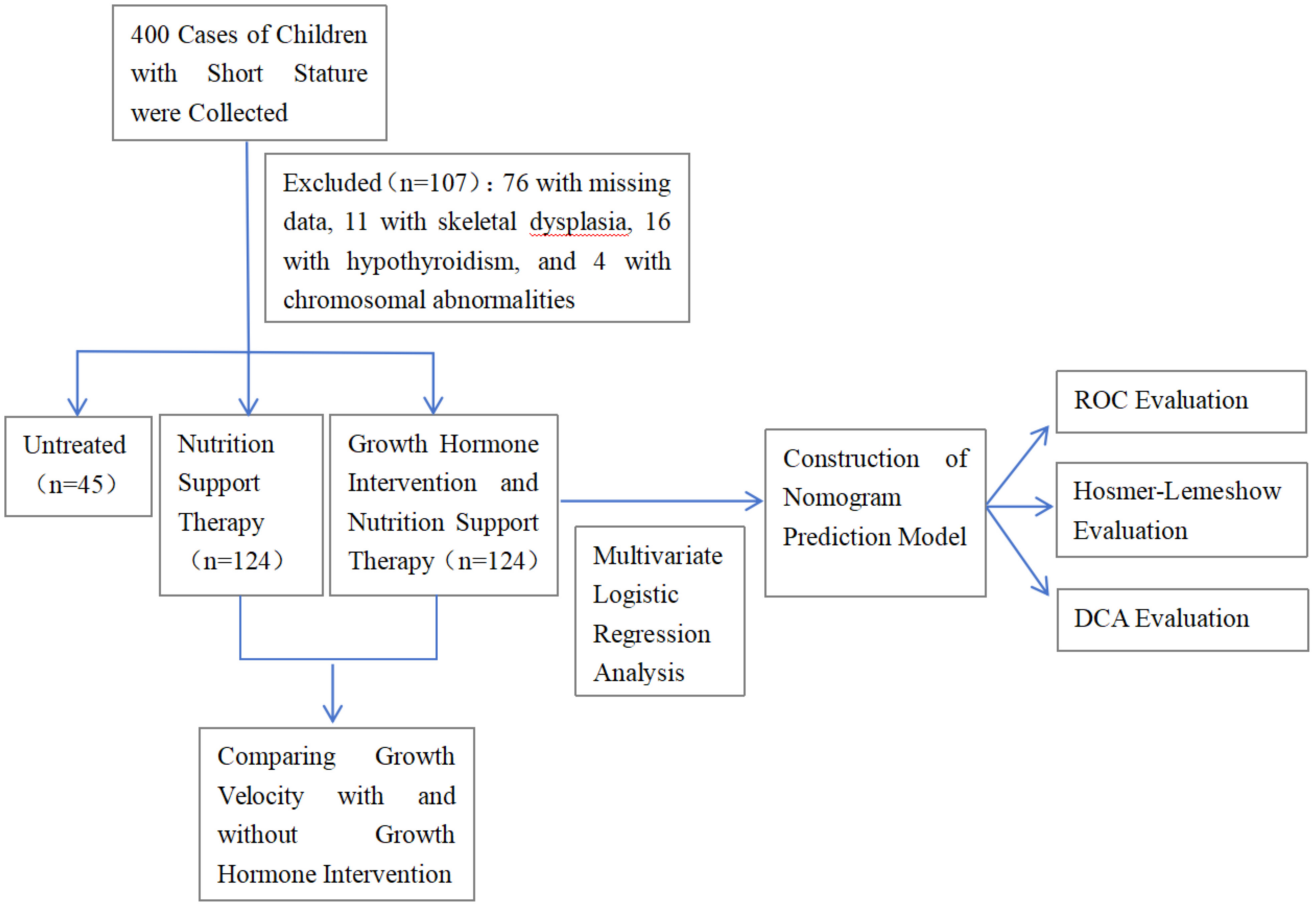
The flowchart of participants in this study. (A total of 400 children with short stature were recruited. Qualified subjects were allocated to three groups. We compared growth velocity among treated groups, analyzed factors associated with growth response in the growth hormone group, established and validated the corresponding prediction model.)

In this study, 400 patients with short stature in 2025 were enrolled. According to the above inclusion and exclusion criteria, after excluding 107 patients (76 with missing data, 11 with skeletal dysplasia, 16 with hypothyroidism, and 4 with chromosomal abnormalities), a total of 293 patients were finally included in the analysis. These comprised 124 patients in the hormone intervention group, 124 in the nutritional support group, and 45 in the untreated group. Figure 1 shows the flowchart of participants in this study and data from 293 participants are summarized in Table 1. Baseline characteristics of the three groups are shown in Supplementary Table 1.

**Table 1:** Information on 293 participants.

Quantitative variables	Mean	SD	Range	Median (P_25_, P_75_)	Qualitative variables		Counts (*n*)	Percent (%)
Age (year)	5.46	2.4	2–14	5.1 (3.8,6)	Gender	Male	176	60.07
Height (cm)	101.55	12.78	75–150.2	100.5 (92.5,106.7)		Female	117	39.93
Weight (kg)	16.15	5.72	8.4–54	15.2 (13, 17)	Age (year)	2	27	9.22
Father's Height (cm)	169.95	5.73	139.9–185	170 (167,173)		3	48	16.38
Mother's Height (cm)	157.55	5.54	120–172	158 (155,160)		4	62	21.16
Target Height (cm)	161.99	7.33	141.15–180	160.5 (156.5,167.88)		5	74	25.26
Birth Weight (kg)	3.02	0.5	1.39–4.3	3 (2.8,3.35)		6	36	12.29
Birth Height (cm)	48.74	2.39	40–52	50 (48,50)		7	9	3.07
HtSDS	−2.6	0.65	−6–−2	−2.381 (−2.8, −2.17)		8	5	1.71
Vitamin D (μg/L)	25.33	11.36	7–87.03	24.5 (18.2, 29.6)		9	7	2.39
Hb (g/L)	128.08	9.58	83–168	130 (124, 135)		10	8	2.73
Glucose (mmol/L)	4.98	0.49	2.23–6.61	5.08 (4.81, 5.31)		11	5	1.71
IGF-1 (μg/L)	261.36	170.53	10.1–884.5	259.46 (94.1, 355.28)		≥12	12	4.10
IGFBP3 (mg/L)	3.62	1.06	0.61–6.1	3.734 (2.94, 4.43)	Full-term or not	Preterm infant	35	11.95
TC (mmol/L)	4.15	0.86	0.54–6.6	4.09 (3.63, 4.63)		Full-term infant	258	88.05
Ca (mmol/L)	1.54	0.10	1.20–1.80	1.55 (1.49, 1.61)	Way of birth	Cesarean section	78	26.62
Zn (μmol/L)	65.78	8.69	49.30–94.70	66 (61.03, 70.85)		Normal delivery	215	73.38

SD: standard deviation; HtSDS: height standard deviation scores; Hb: hemoglobin; IGF-1: insulin-like growth factor-1; IGFBP3: insulin-like growth factor binding protein 3; TC: total cholesterol; Ca: Calcium; Zn: zinc.

**Figure 2:**
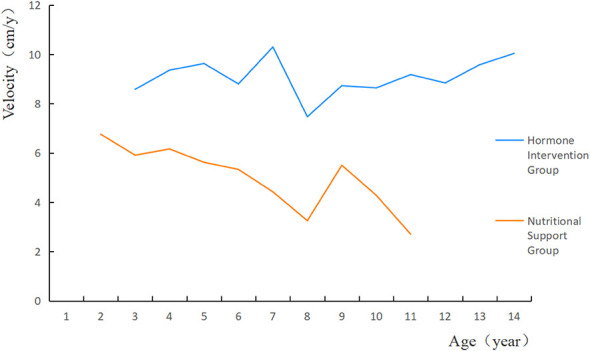
Comparison of annualized growth velocity between the growth hormone intervention group and nutritional support group across different age subgroups of children with short stature.

As presented in Table 1, the participants were aged 2 to 14 years. Males (*n* = 176) accounted for a larger proportion than females (*n* = 117), and most children (87.38%) were younger than 7 years. Analyzing treatment modalities, patients in the hormone intervention group were aged 3 to 14 years old, while those receiving nutritional support therapy alone were aged 2 to 11 years old. Figure 2 illustrates the growth velocity of children in different age groups across the two groups.

As shown in the figure above, although both groups exhibited fluctuations in growth velocity, the hormone intervention group consistently maintained a higher annualized growth velocity than the nutritional support group overall (*p* < 0.05). Furthermore, the growth velocity in the nutritional support group showed a declining trend with increasing age. Between the ages of 3 and 11, the growth velocity in the hormone intervention group exceeded that of the nutritional support group by 2.67–6.48 cm/year. After multivariable adjustment, the difference in annual growth velocity between the two groups remained statistically significant (*p* < 0.001). The annual growth velocity in the growth hormone group was on average 3.66 cm/year higher than that in the nutritional support group (Table 2). This demonstrates that growth hormone therapy is a viable option for treating short stature (14).

**Table 2:** Multiple linear regression analysis for factors associated with annualized growth velocity.

Variables	β	Standard Error	*t* value	*P* value	95% CI
Group (growth hormone intervention vs. nutrition support)	3.664	0.358	10.24	< 0.001	2.959 ~ 4.369
Gender (Female as reference)	−0.215	0.226	−0.95	0.342	−0.661 ~ 0.231
Age	−0.187	0.079	−2.37	0.018	−0.342–0.032
Baseline height	0.029	0.016	1.81	0.072	−0.002 ~ 0.060
SDS	−0.162	0.138	−1.17	0.241	−0.432 ~ 0.108
IGF-1	0.004	0.001	5.72	< 0.001	0.003 ~ 0.005

SDS: standard deviation scores; 95% CI: 95% confidence interval.

### 3.2 Model development and validation

Treatment efficacy on height improvement was evaluated by monitoring ΔHtSDS changes before and after intervention. Demographic characteristics and differential analyses of the treatment-effective group (*n* = 20) and treatment-highly-effective group (*n* = 66) within the training set (*n* = 86) are presented in Table 3.

**Table 3:** Demographic characteristics and differential analysis of the training set population.

Variables	Total (*n* = 86)	Treatment-effective (*n* = 20)	Treatment-highly-effective (*n* = 66)	Statistic	*p*
Age, Mean ± SD	6.34 ± 2.54	7.46 ± 2.77	5.99 ± 2.38	*t* = 2.33	0.022
Height, Mean ± SD	105.99 ± 13.38	111.33 ± 12.40	104.38 ± 13.33	*t* = 2.07	0.041
Weight, Mean ± SD	17.75 ± 5.57	19.49 ± 5.49	17.23 ± 5.52	*t* = 1.61	0.112
Father's Height, Mean ± SD	169.12 ± 6.07	168.83 ± 5.83	169.20 ± 6.18	*t* = −0.23	0.822
Mother's Height, Mean ± SD	157.07 ± 5.66	154.82 ± 7.02	157.76 ± 5.04	*t* = −1.97	0.052
Target Height, Mean ± SD	165.02 ± 7.23	161.77 ± 7.66	166.02 ± 6.85	*t* = −2.24	0.028
Birth Weight, Mean ± SD	3.13 ± 0.46	3.26 ± 0.47	3.09 ± 0.45	*t* = 1.33	0.187
Birth Height, Mean ± SD	49.33 ± 1.64	48.75 ± 2.12	49.58 ± 1.39	*t* = −1.21	0.238
HtSDS, Mean ± SD	−2.67 ± 0.66	−2.50 ± 0.51	−2.72 ± 0.70	*t* = 1.31	0.193
Duration of treatment, Mean ± SD	2.11 ± 1.19	1.24 ± 1.01	2.37 ± 1.12	*t* = −4.05	< 0.001
Vitamin D, Mean ± SD	21.87 ± 8.13	20.04 ± 5.96	22.41 ± 8.64	*t* = −1.02	0.310
Hb, Mean ± SD	131.61 ± 10.45	132.94 ± 4.93	131.21 ± 11.62	*t* = 0.58	0.566
Glucose, Mean ± SD	5.20 ± 0.31	5.24 ± 0.23	5.18 ± 0.33	*t* = 0.75	0.457
IGF-1, Mean ± SD	377.86 ± 136.23	304.32 ± 113.54	400.15 ± 135.40	*t* = −2.87	0.005
IGFBP3, Mean ± SD	4.32 ± 1.06	4.60 ± 0.78	4.24 ± 1.13	*t* = 0.90	0.373
TC, Mean ± SD	4.24 ± 0.81	4.07 ± 0.80	4.29 ± 0.81	*t* = −0.96	0.340
Ca, Mean ± SD	1.43 ± 0.18	1.42 ± 0.12	1.44 ± 0.21	*t* = −0.19	0.858
Zn, Mean ± SD	64.75 ± 8.59	59.17 ± 4.79	67.14 ± 8.98	*t* = −1.42	0.193
Gender, *n* (%)				χ^2^ = 4.06	0.044
Male	55 (63.95)	9 (45.00)	46 (69.70)		
Female	31 (36.05)	11 (55.00)	20 (30.30)		
Full-term or not, *n* (%)				χ^2^ = 0.02	0.890
Preterm infant	10 (11.63)	3 (15.00)	7 (10.61)		
Full-term infant	76 (88.37)	17 (85.00)	59 (89.39)		
Way of birth, *n* (%)				χ^2^ = 0.02	0.885
Cesarean section	16 (18.60)	3 (15.00)	13 (19.70)		
Normal delivery	70 (81.40)	17 (85.00)	53 (80.30)		

*T, t*-test; χ^2^, Chi-square test.

We used VIF to test multicollinearity of age, height, target height and IGF-1 (VIF = 3.46, 19.11, 1.11, 4.12). The moderate collinearity between age and height is a natural physiological trait in children and does not affect the regression model. Univariate analysis in the training set showed that age, baseline height, target height, treatment duration, IGF-1, and gender were associated with treatment efficacy (*p* < 0.05). Logistic regression further verified the significant association between treatment duration and therapeutic response (*p* = 0.006). Results are shown in Table 4.

**Table 4:** Multivariate logistic regression for treatment efficacy.

Variables	β	S.E	Z	*p*	OR (95%CI)
Gender					
Male					1.00 (Reference)
Female	−1.00	0.82	−1.23	0.221	0.37 (0.07 ~ 1.82)
Age	−0.56	0.56	−1.00	0.316	0.57 (0.19 ~ 1.71)
Height	0.03	0.11	0.26	0.791	1.03 (0.84 ~ 1.27)
Target Height	0.08	0.06	1.44	0.151	1.08 (0.97 ~ 1.21)
Duration of treatment	1.49	0.54	2.75	0.006	4.45 (1.53 ~ 12.93)
IGF-1	0.01	0.00	1.41	0.157	1.01 (1.00 ~ 1.01)

**Figure 3:**
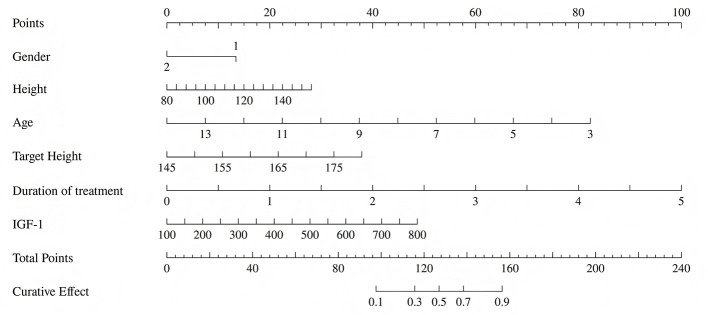
Nomogram prediction model for evaluating the efficacy of growth hormone therapy in children with short stature. Six clinical variables were incorporated to predict treatment response.

To visually assess the response of each factor to treatment, the six optimal predictors were incorporated into a nomogram model (Figure 3). This nomogram employs a weighted scoring system to quantify each factor, with a maximum score of 240 points. Analysis revealed that duration of hormone therapy most significantly influenced growth velocity in pediatric patients, followed by age at initial treatment, serum IGF-1 levels, target height, and height at initial treatment. Gender also influences treatment response, but to a relatively lesser extent.

**Figure 4:**
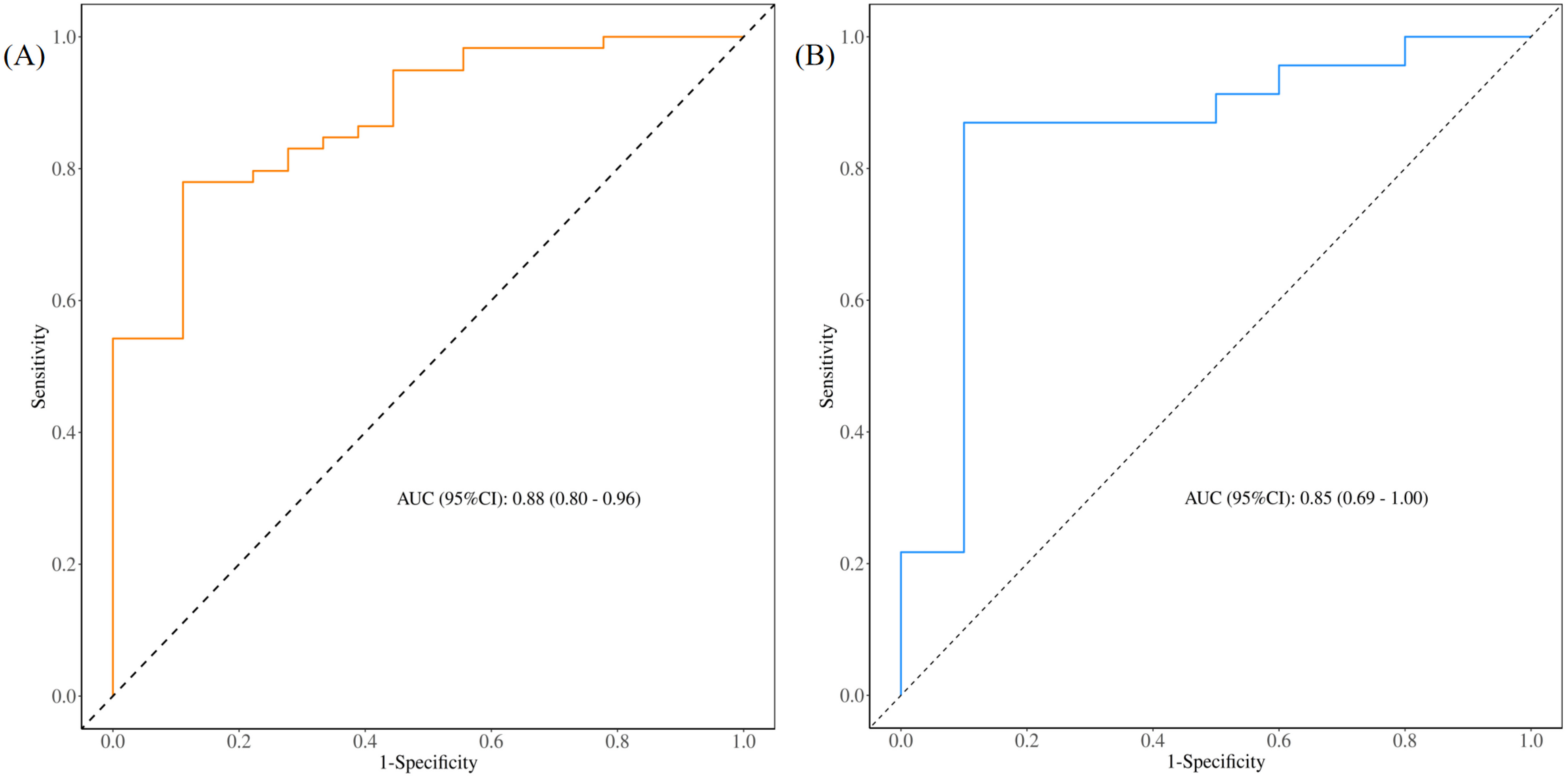
Receiver operating characteristic (ROC) curves for assessing the discriminative ability of the nomogram model. **(A)** ROC curve of the training set; **(B)** ROC curve of the internal validation set. AUC values represent the predictive performance of the model.

**Figure 5:**
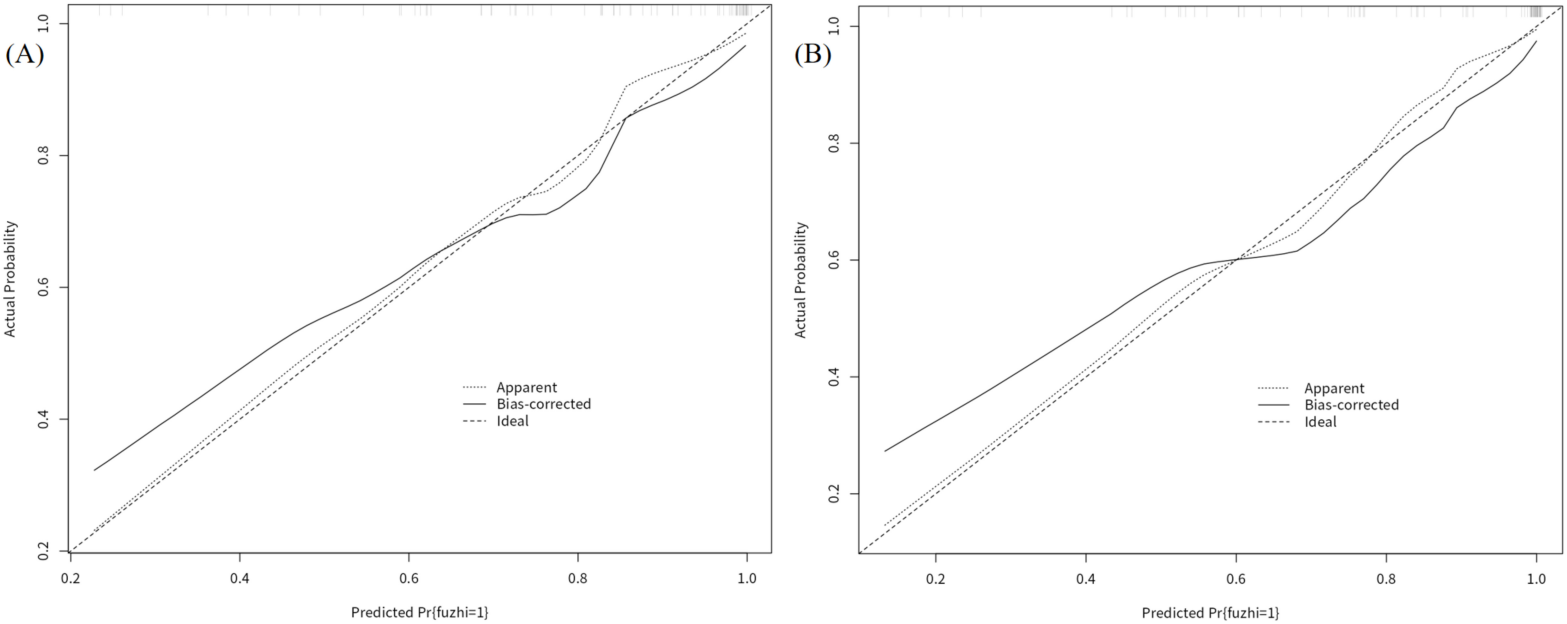
Calibration curves of the established nomogram model. **(A)** Calibration curve for the training set; **(B)** Calibration curve for the internal validation set. The closer the solid line fits the diagonal dashed line, the better the consistency between predicted and actual outcomes.

The discrimination and precision of the nomogram model were evaluated using ROC and calibration curves. The area under the curve (AUC) value for the training set was 0.88 (95% confidence interval (CI): 0.80–0.96), while the validation set yielded an AUC of 0.85 (95% CI: 0.69–1.00) (Figures 4A, B), indicating that the predictive model possesses good discriminatory capability. Calibration curve analysis (Figures 5A, B) shows the predicted probability of efficacy on the horizontal axis and the actual occurrence probability on the vertical axis. The diagonal dotted line represents the perfect prediction performance of an ideal model, the solid line represents the results of the nomogram. A closer fit between the solid line and the diagonal dashed line indicates higher predictive accuracy and favorable agreement between predicted and actual outcomes. The Hosmer-Lemeshow test revealed *p*-values of 0.679 and 0.856 for the training and validation sets, respectively (the model passes the test when *p*-values > 0.05), indicating good model fit.

**Figure 6:**
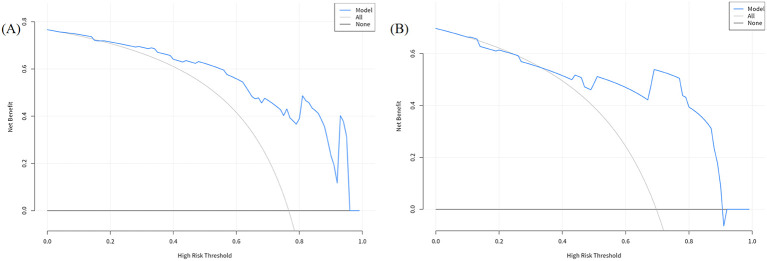
Decision curve analysis (DCA) for evaluating the clinical net benefit of the nomogram model. **(A)** DCA curve of the training set; **(B)** DCA curve of the internal validation set. The curves demonstrate the clinical application value of this prediction model.

The clinical utility of this model was further evaluated using decision curve analysis (DCA) curves (Figures 6A, B). The horizontal axis represents threshold probability, while the vertical axis displays net benefit. The black horizontal solid line represents the assumption of no intervention is administered in all children. The gray solid line corresponds to the net benefit, while the blue solid line depicts the decision curve. Analysis indicates that when the threshold probability in the training set falls below 96% and in the validation set below 91%, the model still predicts a positive net benefit for treatment efficacy. This indicates that the model possesses high practical value in predicting the efficacy of hormone therapy.

## 4 Discussion

With rising living standards, children's height has generally increased, yet concerns about short stature persist (15, 16). This study found male pediatric patients to be more common than female patients, consistent with previous domestic research (17). Short stature is more common in male pediatric patients than in female patients. This gender disparity is mainly due to the higher prevalence of GHD in males, greater clinical referral bias for short stature in boys, and the higher frequency of idiopathic and familial short stature among male children. In addition, males have a later onset of puberty, leading to slower prepubertal growth and earlier clinical recognition of short stature. Compared with conventional nutritional support, growth hormone therapy is an effective intervention for children with short stature of diverse etiologies including GHD and ISS, and it has been widely used clinically for many years (18). Growth hormone treatment was associated with significantly higher annualized growth velocity compared with nutritional support alone. Meanwhile, we observed that the age distribution of the growth hormone intervention group was shifted to the right compared with the nutritional support group. It is speculated that parents of younger children tend to prefer conservative expectant treatment; as age increases, parents become increasingly concerned about their children's growth potential and therefore choose growth hormone therapy. Furthermore, considering the cost of long-term growth hormone treatment, this explains why some families only choose nutritional support therapy.

A prediction model for growth hormone efficacy was constructed and internally validated. Notably, duration of growth hormone therapy was the only independent significant predictor. This finding is consistent with clinical evidence that longer treatment duration is associated with better height gain. However, since treatment duration is observed during therapy rather than at baseline, the model is more appropriate for intra-treatment efficacy evaluation and follow-up optimization rather than pre-treatment decision making.

The nomogram demonstrated that longer treatment duration and younger age at treatment onset were linked to a higher likelihood of favorable outcomes. This trend is supported by previous clinical findings regarding long-term growth hormone intervention (19, 20). This highlights the critical importance of early intervention. After favorable height gains were achieved in younger children with treatment, combined with the economic burden of long-term therapy, this explains the lower proportion of older children presenting for hospital care shown in Table 1.

Growth and development is a complex process involving multiple factors such as genetics, nutrition, and hormones. The growth hormone/insulin-like growth factor-1 axis, a key regulatory pathway governing childhood growth, plays a central role during critical developmental stages (21–24). As a mediator of growth hormone, IGF-1 promotes growth by directly acting on target organ (25). This suggests that regular monitoring of IGF-1 levels is essential during treatment. Higher IGF-1 levels indicate better therapeutic response and can serve as a monitoring indicator for timely adjustment of growth hormone dosage (20, 26).

Formulas exist to predict a child's target adult height based on parental height (13), but few studies have examined the relationship between target height and responsiveness to growth hormone therapy. Our model revealed that higher target height correlates with higher scores on the growth chart, indicating a greater likelihood of high responsiveness to growth hormone therapy. This indirectly suggests that children of taller parents may exhibit higher growth rates during growth hormone treatment (7, 19).

Furthermore, we observed that initial height at the start of treatment influences the efficacy of growth hormone therapy. Among children with short stature in the same age group, taller children demonstrate greater growth potential in response to growth hormone treatment, while shorter children show poorer outcomes. Gender also exhibits a certain degree of association with treatment responsiveness. In this nomogram, males achieved slightly higher scores than females, suggesting boys may exhibit greater responsiveness. The findings on how height and gender influence the response to growth hormone therapy are in line with those reported in prior literature (19, 27).

This study has several limitations. The study population presented heterogeneous causes of short stature. Given the small sample size, subgroup analyses based on etiology and pubertal status were not conducted. As a single-center retrospective study, external validation was not performed. Treatment allocation was based on parental preference rather than randomization. In the future, we will increase the sample size to avoid overfitting, and conduct stratified analyses by etiology and pubertal status. Larger independent cohorts will be adopted for multicenter external validation to acquire more reliable findings and refine the predictive model.

## 5 Conclusions

Growth hormone effectively increases annual growth velocity in children with short stature, and treatment duration plays a critical role in determining the final therapeutic outcome. A prediction model incorporating six clinical variables demonstrated good discriminatory ability and calibration. Due to several limitations, we aim to further optimize the protocol in future studies so that it may assist clinicians in performing individualized dynamic efficacy assessments and subsequent management, with the potential to optimize follow-up treatment strategies.

## Data Availability

The raw data supporting the conclusions of this article will be made available by the authors, without undue reservation.
