# Supplementary material for: Population characteristics of children with short stature and construction of a predictive model for growth hormone treatment efficacy
Source: Front Med (Lausanne). 2026 Jul 7;13:1818279. doi: 10.3389/fmed.2026.1818279 (PMC13384815; doi:10.3389/fmed.2026.1818279)
Supplement: Supplementary file 1 [file Table_1.doc]

Supplementary Table 1. Baseline characteristics of participants in the three groups

| **Characteristic** | **Untreated group (n=45)** | **Nutrition support group (n=124)** | **Growth Hormone treatment group (n=124)** | **P value** |
| --- | --- | --- | --- | --- |
| Age（year） | 5.07 ± 2.20 | 4.61 ± 1.70 | 6.46 ± 2.70 | <0.0001 |
| Height（cm） | 100.17 ± 12.71 | 97.06 ± 9.89 | 106.54 ± 13.64 | <0.0001 |
| Weight（kg） | 16.24 ± 7.42 | 14.11 ± 3.60 | 18.15 ± 6.07 | <0.0001 |
| Target Height （cm） | 165.48 ± 7.53 | 165.75 ± 7.76 | 165.23 ± 7.45 | 0.6682 |
| Father's Height（cm） | 169.84 ± 5.30 | 170.15 ± 6.06 | 169.80 ± 5.60 | 0.5447 |
| Mother's Height（cm） | 159.00 ± 4.76 | 157.72 ± 4.52 | 156.79 ± 6.59 | 0.1725 |
| Target Height （cm） | 165.48 ± 7.53 | 165.75 ± 7.76 | 165.23 ± 7.45 | 0.6682 |
| Height SDS | -2.48 ± 0.68 | -2.57 ± 0.62 | -2.66 ± 0.67 | 0.0886 |
| Vitamin D（μg/L） | 23.86 ± 11.47 | 25.24 ± 12.68 | 22.61 ± 8.00 | 0.8592 |
| Hb（g/L） | 125.05 ± 8.55 | 127.26 ± 8.31 | 128.03 ± 9.86 | 0.1389 |
| Glucose （mmol/L） | 4.83 ± 0.41 | 4.88 ± 0.57 | 4.97 ± 0.35 | 0.0757 |
| IGF-1（μg/L） | 260.12 ±161.41 | 257.22 ± 132.26 | 265.34 ± 159.78 | 0.0530 |
| IGFBP3 （mg/L） | 3.22 ± 1.01 | 3.37 ± 0.90 | 3.10 ± 0.97 | 0.1327 |
| TC（mmol/L) | 4.22 ± 0.95 | 4.11 ± 0.89 | 4.16 ± 0.78 | 0.9553 |
| Ca（mmol/L) | 1.55 ± 0.08 | 1.55 ± 0.09 | 1.46 ± 0.16 | 0.0769 |
| Zinc（μmol/L) | 63.91 ± 8.36 | 66.49 ± 8.98 | 65.29 ± 7.30 | 0.2814 |

# Abbreviations: HtSDS: height standard deviation scores; Hb: hemoglobin; IGF-1: insulin-like growth factor-1; IGFBP3: insulin-like growth factor binding protein 3; TC: total cholesterol; Ca:Calcium; Zn: zinc

There were statistically significant differences in baseline age, height and weight levels among the three groups. This is mainly because the present study is a retrospective clinical investigation without random grouping. In clinical practice, physicians and parents tend to have concerns about the growth potential of older children, and thus prefer growth hormone intervention for them. By contrast, younger children are mostly managed with nutritional support or regular follow-up. Such differences in clinical treatment choices ultimately resulted in higher baseline age and physical growth indicators in the growth hormone treatment group compared with the nutritional support group and the untreated group. These discrepancies occurred naturally during clinical enrollment. Since the remaining baseline characteristics were well balanced across groups, the reliability of the research results was not compromised.
